# Modulation of cardiometabolic pathways in skin and serum from patients with psoriasis

**DOI:** 10.1186/1479-5876-11-194

**Published:** 2013-08-22

**Authors:** Nehal N Mehta, Katherine Li, Philippe Szapary, James Krueger, Carrie Brodmerkel

**Affiliations:** 1 Section of Inflammation and Cardiometabolic Diseases, National Heart Lung and Blood Institute, Bethesda, MD, USA; 2 Janssen Research & Development, LLC, Spring House, PA, USA; 3 Rockefeller University, New York, NY, USA

**Keywords:** Inflammation, Atherosclerosis, Lipid metabolism, Psoriasis

## Abstract

**Background:**

Moderate-to-severe psoriasis is associated with an increased risk of atherosclerotic cardiovascular disease (ASCVD); however, the link is poorly understood.

**Methods:**

Skin and serum from patients with psoriasis were evaluated to understand if there was evidence of dysregulation in a targeted group of inflammatory and lipid genes related to ASCVD. Microarray analyses of expression of targeted ASCVD genes from skin in 89 patients with moderate-to-severe psoriasis from the ACCEPT trial were compared with non-diseased skin from healthy controls (n = 25). Serum (n = 149) was tested at baseline for monocyte chemoattractant protein-1 (MCP-1), macrophage-derived chemokine (MDC), and apolipoprotein-A1 (Apo-A1) comparing to healthy controls (n=162).

**Results:**

An increase in skin gene expression for MCP-1 (7.98-fold) and MDC (6.66-fold) (p < 0.001 each) was observed in lesional versus healthy skin. Significant decreases in liver X receptor-alpha (LXR-α) (−5.94-fold), a protective lipoprotein metabolism gene, and in peroxisome proliferator-activated receptor-alpha (PPAR-α) (−7.58-fold), a protective anti-inflammatory and lipid modulating gene, were observed in lesional versus healthy skin (p < 0.001 each). Serum analyses revealed that MCP-1 (502 vs. 141 pg/mL) and MDC (1240 vs. 409 pg/mL) levels were significantly elevated in psoriasis compared with healthy controls (p < 0.001 each). Dysregulated lipid metabolism was also evident in the serum, as Apo-A1, a protein product related to PPAR-α activation, was significantly decreased in patients with psoriasis compared with healthy controls (25.2 vs. 38.9 mg/dL; p < 0.001).

**Conclusions:**

Analyses of targeted genes and their products known to be associated with ASCVD revealed dysregulation of inflammatory (MCP-1 and MDC) and lipid metabolism (LXR-α, PPAR-α) genes in psoriasis. These findings provide evidence of a potential shared pathophysiology linking psoriasis to cardiometabolic diseases.

## Background

Psoriasis is a chronic, inflammatory skin disease that places some patients at increased risk of myocardial infarction [1], stroke [2], cardiovascular (CV) death [3], overall mortality [4], and other major adverse CV events [5]. The cause for this increased CV risk in psoriasis remains unknown, although evidence suggests shared inflammatory pathways in psoriasis and atherosclerosis [6]. Macrophage-derived chemokine (MDC) and monocyte chemotactic protein-1 (MCP-1) are involved in the trafficking of activated T-lymphocytes to inflammatory sites. MDCs and MCP-1 are strongly chemotactic for monocytes, dendritic cells, and natural killer cells, which are involved in atherosclerosis and psoriasis, providing a potential link to these two chronic diseases [7]. The skin is a lipid-rich organ which expresses nuclear hormone receptors including liver X receptor (LXR) and peroxisome proliferator-activated receptor (PPAR), which may be dysregulated in psoriasis [8]. LXR is a key regulator of reverse cholesterol transport of lipids from atherosclerotic plaques back to the liver [9] and is important in inflammation and insulin resistance. PPARs are important in lipid metabolism [10]. PPAR-α is the target of fibrate drugs that raise high density-lipoprotein (HDL), as well as production of apolipoprotein A1 (Apo-A1), the major lipoprotein of HDL [11]. Building on our observations from pathway analysis of lesional versus nonlesional skin gene expression [12], we selected genes related to atherosclerotic cardiovascular disease (ASCVD) to evaluate expression of these select genes in the skin and serum from patients with psoriasis. We hypothesized that ASCVD genes related to inflammation in lesional skin from patients with moderate-to-severe psoriasis may lead to systemic manifestation of inflammation by dysregulated expression of the inflammatory and lipoprotein metabolism proteins in the serum.

## Methods

### Populations studied

ACCEPT was a Phase 3, multicenter, randomized, active-controlled, parallel, 3-arm study of patients with moderate-to-severe plaque psoriasis to investigate the effects of an anti-IL-12/23p40 mAb (ustekinumab) compared to anti-TNF therapy (etanercept) [13]. Here, we compare serum analyses and expression of ASCVD-related genes between psoriatic skin (lesional and macroscopically normal nonlesional skin) in patients from the ACCEPT trial who underwent skin biopsies versus two separate healthy, non-psoriatic control populations (one for skin analyses [n = 25] and one for serum analyses [n = 162]). Microarray data from the biopsy substudy of the ACCEPT trial [13] were used for comparison of baseline lesional and nonlesional skin in 89 psoriasis patients from the ACCEPT trial to healthy skin (n = 25). Skin samples from 25 healthy subjects (controls) who were selected to match in age and gender with the psoriatic cohort in the skin biopsy analysis were obtained from Rockefeller University (New York, NY, USA) under institutional review board-approved written informed consent. Serum samples were obtained from 162 healthy volunteers (Bioreclimation, Hicksville, NY, USA) with written informed consent and a subset of 149 patients with psoriasis from the ACCEPT trial. There was not a complete overlap of the patients assessed for serum protein expression and skin biopsy analysis in the ACCEPT trial. Of the 149 patients with psoriasis assessed for serum protein expression, 62 were also part of the skin biopsy substudy and this was a randomly selected subset. Patients with psoriasis did not have any clinical CV disease.

### Skin biopsy analysis

Skin biopsies were isolated from a representative psoriatic target lesion (3 cm) and identified as lesional psoriatic skin. Nonlesional skin biopsies were obtained from a similar body area to the psoriasis lesion (i.e., arm, arm or leg, leg) in the same method as lesional skin was obtained. RNA was extracted with the RNeasy Fibrous Tissue Mini Kit (QIAGEN Inc., Valencia, CA, USA). Microarray hybridization was conducted using GeneChip HG-U133 Plus 2.0 arrays (Affymetrix, Santa Clara, CA, USA).

### Serum analysis

Serum (n = 149; including 62 patients from the biopsy substudy) was tested at baseline for cytokines and chemokines (MCP-1 and MDC), C-reactive protein (CRP), and plasma Apo-A1 using a Luminex-based platform (HumanMAP®, Myriad RBM, Inc., Austin, TX, USA). Sixteen of the 43 candidate genes had protein products measured in serum, including CD40-ligand, myeloperoxidase, MCP-1, PAI1, MDC, TNF-α, IL-18, leptin, TNF-β, Apo-A1, IFN-γ, apolipoprotein-CIII, MMP-9, IL-6, PAPPA, and adiponectin. Raw data were log-transformed to achieve normal distribution of the data prior to analyses.

### Statistical analyses

Summary statistics were used to describe sample demographics. Normalization, quality control, and analysis of the microarray data were conducted using Array Studio v6.0 (OmicSoft Corporation, Cary, NC, USA). Gene expression data were normalized by GC-RMA and transformed by log2 scale for data analysis. Microarray data quality was assessed by Pearson’s correlation and Principle Component Analysis. Significant modulations of the targeted genes related to ASCVD between paired lesional and nonlesional samples were identified by general linear model. Subject was included in the model as a random factor. Differences in serum protein levels were tested using t-tests for normally distributed variables and by Kruskal-Wallis testing for non-normally distributed variables. Multiple testing correction (false discovery rate <5%) was applied to both microarray and serum analyses. We examined the correlation of baseline psoriasis disease severity as measured by Psoriasis Area and Severity Index (PASI) score and serum protein expression using Spearman’s correlation.

## Results

### Baseline demographics and disease characteristics

Baseline demographics and disease characteristics of the patients with psoriasis are shown in Table 1. In addition, 162 healthy control subjects, of similar age, race, sex, and mostly free of CV disease risk factors, were assessed for serum protein expression.

**Table 1:** Baseline demographic and disease characteristics of patients with psoriasis

Randomized patients, n	149
Age, years, Mean ± SD	46.3 ± 12.8
Sex	
Men, n (%)	114 (76.5)
White, n (%)	133 (89.3)
Psoriasis body surface area involvement, Mean ± SD	29.5 ± 18.6
Psoriasis Area and Severity Index (PASI) score, Mean ± SD	21.3 ± 9.0
Psoriatic arthritis, n (%)	38 (25.5)
Body Mass Index (BMI), n (%)	
N	148
Normal (BMI <25)	29 (19.6)
Overweight (BMI ≥25 and <30)	46 (31.1)
Obese (BMI ≥30)	73 (49.3)
Metabolic Syndrome, n (%)^a^	41 (27.5)
Cigarette smoking, n (%)	
Current	50 (33.6)
Past or current	97 (65.1)
Relevant medical history, n (%)	
ASCVD^b^	6 (4.0)
Diabetes mellitus	21 (14.1)
Hypertension	41 (27.5)
Hyperlipidemia	31 (20.8)
Family history of early ASCVD	15 (10.1)
High-density lipoprotein (HDL), n	141
Mean ± SD	47.9 ± 12.9
HDL <40 mg/dL, n (%)	43 (30.5)
Low-density lipoprotein, n	137
Mean ± SD	113.1 ± 35.7
Triglycerides, n	141
Median (IQ range)	134.0 (99.0, 182.0)
Total Cholesterol, n	141
Mean ± SD	191.8 ± 40.0
Total Cholesterol/HDL, Mean ± SD	4.3 ± 1.4

*ASCVD* atherosclerotic cardiovascular disease, *IQ* interquartile range.

^a^Metabolic syndrome is defined using modified National Cholesterol Education Program criteria (waist circumference was replaced with BMI ≥ 30) including the presence of 3 or more of the 5 risk factors: BMI ≥ 30; fasting triglycerides ≥ 150 mg/dL; fasting HDL < 40 mg/dL (male) or HDL < 50 mg/dL (female); baseline systolic pressure ≥ 130 mmHg, baseline diastolic pressure ≥ 85 mmHg, or treatment for hypertension; fasting glucose ≥ 100 mg/dL

^b^ASCVD is defined as having a baseline medical history of myocardial infarction, coronary artery bypass, stroke, transient ischemic attack, or peripheral vascular disease

### Gene expression of targeted ASCVD genes related to lipid metabolism and inflammatory genes differs between healthy skin, psoriatic nonlesional and lesional skin

**Figure 1:**
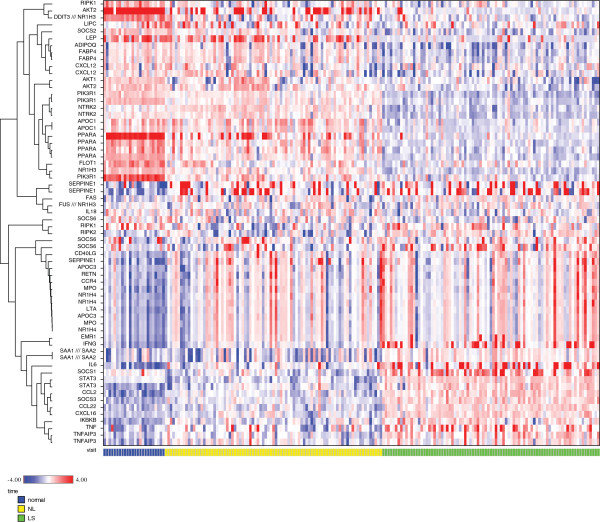
**Expression profile of 43 targeted cardiovascular, metabolic, and inflammatory-related genes in healthy normal, nonlesional, and lesional skin samples at baseline.** CCL2 = MCP-1; CCL22 = MDC; NR1H3 = LXR.

**Figure 2:**
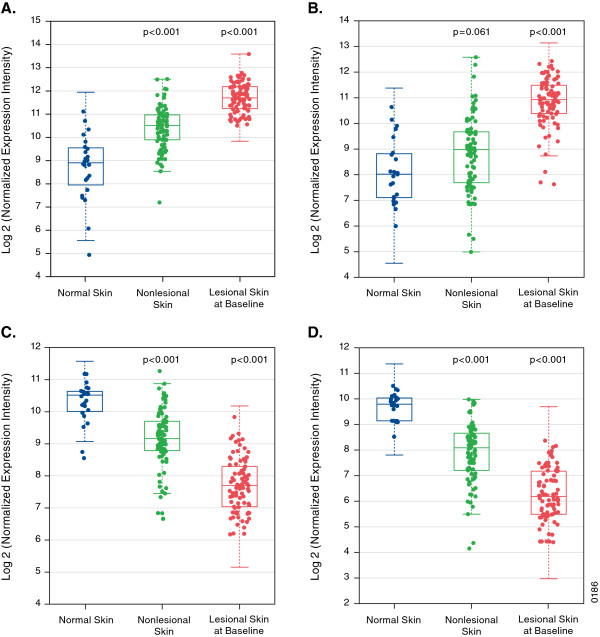
**Microarray skin expression of (A) monocyte chemotactic protein-1 (MCP-1), (B) macrophage-derived chemokine (MDC), (C) liver X receptor-alpha (LXR-α), and (D) peroxisome proliferator-activated receptor-alpha (PPAR-α) between healthy controls (normal skin) and patients with psoriasis (lesional and nonlesional skin) at baseline.** The array contains specific isoforms of genes depicted.

Genes linked to cardiometabolic pathways were assessed by microarray for expression. A total of 43 genes chosen *a priori* to be related to the pathophysiology of atherosclerosis, metabolic, or inflammatory dysregulation were assessed in normal, nonlesional, and lesional skin samples. Of the 43 selected genes, 24 were differentially expressed at a nominal level in psoriasis lesions compared with healthy controls (Additional file 1: Table S1). The heatmap (Figure 1) demonstrated a pattern for a cluster of lipid metabolism-related genes that were upregulated in healthy skin, downregulated in nonlesional skin, and further downregulated in lesional skin. The opposite was observed for a cluster of inflammation-related genes. In particular, two chemotactic genes involved in psoriasis and atherosclerosis (MCP-1 and MDC) were upregulated in comparisons between lesional and normal skin and between nonlesional and normal skin. LXR-α and PPAR-α expression was downregulated (Figure 2). No correlation between the PASI score and MCP-1 or MDC expression was observed. However, there was negative correlation of PASI score with PPAR-α (r = −0.27, p = 0.013) and Apo-A1 (r = −0.23, p = 0.034) expression, and no correlation between PASI and MCP-1 (r = 0.13, p = 0.23) expression.

### Protein levels of inflammatory, chemotactic, and lipoproteins differ between patients with psoriasis and healthy controls

**Figure 3:**
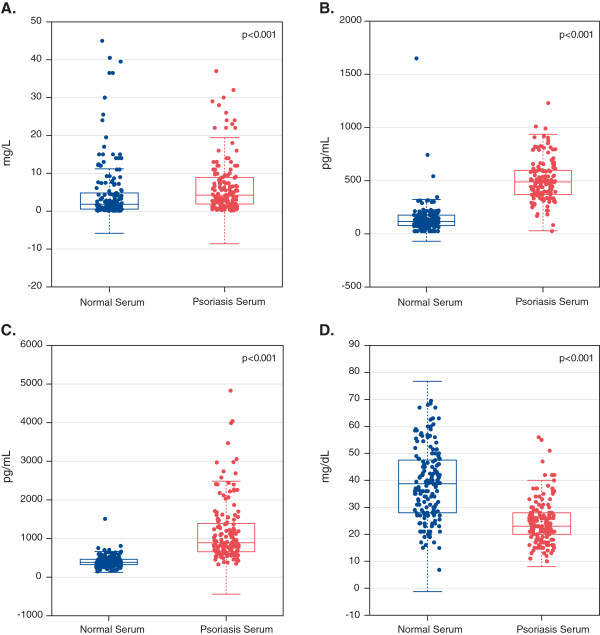
Microarray serum expression of (A) C-reactive protein (CRP), (B) monocyte chemotactic protein-1 (MCP-1), (C) macrophage-derived chemokine (MDC), and (D) apolipoprotein-A1 (Apo-A1) between healthy controls (normal serum) and patients with psoriasis (psoriasis serum) at baseline.

Serum samples were analyzed to evaluate if targeted genes associated with CV disease in the skin were also associated with pathways in the blood (Figure 3). Sixteen of the 43 genes had protein products measured in serum. Among them, 10 of the 16 were differentially expressed at a nominal level (Additional file 1: Table S2). CRP levels were higher in patients with psoriasis compared with controls (mean CRP 6.68 mg/L vs. 4.97 mg/L; p < 0.001). Proteins associated with CV disease, MCP-1 (502 pg/mL vs. 141 pg/mL) and MDC (1240 pg/mL vs. 409 pg/mL), were elevated in patients with psoriasis compared with controls (p < 0.001 each). Finally, we observed evidence of dysregulated lipid metabolism, as indicated by a decrease in Apo-A1 in patients with psoriasis (25.2 mg/dL), compared with controls (38.9 mg/dL; p < 0.001). When evaluating if psoriasis disease severity (PASI score) was associated with inflammatory-related and CV disease-related proteins in the blood, it was noted that PASI strongly correlated with serum levels of MDC (r = 0.59, p < 0.001) and CRP (r = 0.27, p = 0.001). There was a weak, non-significant, relationship between PASI and MCP-1 levels in the blood (r = 0.14, p = 0.08), with no significant correlation with Apo-A1 (r = −0.07, p = 0.34).

## Discussion

We report that a targeted group of genes related to ASCVD (MCP-1, MDC, LXR-α, and PPAR-α) are dysregulated in the skin of patients with psoriasis. Corresponding proteins and downstream products (MCP-1, MDC, Apo-A1) also were modulated in the serum. Lesional psoriatic skin demonstrated greater dysregulation of these pathways compared with nonlesional psoriatic skin and normal control skin. Additionally, corresponding serum analyses of MCP-1, MDC, and Apo-A1 were modulated in expected directions consistent with ASCVD findings, suggesting that abnormal skin function may be a potential link between psoriasis and atherosclerosis.

Our findings are consistent with a recent report [14] demonstrating modulation of genes involved in inflammation and lipid metabolism. In addition to examining serum from patients with psoriasis, we also analyzed inflammatory genes associated with CV diseases [15]. Induction of MCP-1 and MDC in psoriatic skin occurred in a linear increase, with healthy skin showing no expression, nonlesional psoriatic skin having some expression, and psoriatic lesional skin having the highest expression. Interestingly, we observed significant downregulation of LXR-α and PPAR-α, genes involved in atherosclerosis, inflammation, and HDL metabolism, which we hypothesized *a priori* would be modulated in psoriatic skin. LXR-α regulates ABCA1 (ATP-Binding Cassette, subfamily A, member 1) and ABCG1, two key transporters of cholesterol in atherosclerotic lesions [9] and inflammatory pathways downstream of nuclear factor kappa-B [10]. PPAR-α regulates the expression of genes involved in cell proliferation, lipid metabolism, and inflammation, including Apo-A1, ABCA1, and HDL.

Prospective cohort and small cross-sectional studies have demonstrated that psoriasis is associated with lower HDL levels [16]. Based on animal models, decreased concentration of HDL due to systemic inflammatory suppression leads to decreased hepatic production of Apo-A1. Our findings suggest that skin inflammation may be involved in the suppression of serum HDL and Apo-A1 concentrations, given that PPAR-α and LXR-α are downregulated in lesional and nonlesional skin of patients with psoriasis and that this was observed to a greater extent in patients with more severe skin involvement. We observed that as disease severity increased, there was greater downregulation of PPAR-α gene expression in the skin.

Our preliminary examination of the systemic circulation corroborated the previous report of complex two-way interactions between activated dendritic cells, T-cells, and keratinocytes mediated by blood cytokines and chemokines [14]. Corresponding to the increased gene expression of MCP-1 and MDC, we observed a significant increase in these gene products in the serum related to disease severity (i.e., PASI). Levels of these proteins are associated with CV disease [17] and are integrally involved in trafficking inflammatory cells to areas of vascular inflammation [18], a phenotype that is enriched in psoriasis [19].

Additionally, we confirmed the hypothesized decrement in serum Apo-A1 levels in psoriasis compared with controls, which corresponded to decreased gene expression in lesional and nonlesional skin. Apo-A1 is involved in reverse cholesterol transport, which was recently demonstrated to be decreased in psoriasis [20]. This association of decreased gene expression of LXR-α and PPAR-α may provide a potential mechanistic insight linking low HDL levels, psoriasis, and ASCVD.

Our study has several limitations. We cannot conclude that inflammation and dysregulation of metabolic genes in the lesional skin are primarily driving the CV risk in patients with psoriasis. However, the findings suggest that modulating known CV and metabolic genes in the skin may in part explain the observed increased risk of ASCVD in severe psoriasis. Furthermore, we do not have atherosclerotic plaque microarray gene expression from patients with psoriasis to complete our explanatory model. Because access to atherosclerotic plaque in a subclinical CV disease population is difficult, our data rely on serum proteins as surrogate markers to deduce the effect of skin on atherosclerosis. We also did not have specific CV demographic profiles for healthy subjects who provided serum for the study. We are limited by the lack of complete demographic data for the healthy serum samples, namely BMI, given the high prevalence of obesity in the patients with psoriasis. However, it is unlikely that these differences are driving the observed differences in protein levels due to the low correlation rate between BMI and the four protein levels of interest (data not shown). Our clinical trial may not be generalizeable to the larger psoriasis population; however, this study includes the largest population of patients with moderate-to-severe psoriasis with characterization of skin gene expression with simultaneous serum analytes important in CV disease. Confirmatory studies of skin microarray gene expression with RT-PCR analysis were not performed as others had reported [14]; however, they reported a high concordance between microarray and real-time PCR, both in psoriasis [14] and in other studies [21,22]. In addition, one control sample population was used for skin biopsy analyses and the other control population was utilized for serum samples. We did not have concurrent samples in the control population.

Here we provide evidence of gene dysregulation within the skin and serum in patients with psoriasis, supporting a shared pathophysiology of psoriasis and ASCVD [23]. Future studies may help to address an *in vivo* link between psoriasis and vascular inflammation using imaging, and by focusing on the effect of psoriasis treatment on CV biomarkers.

## Competing interests

This study was funded by Janssen Research & Development, LLC. N. Mehta has no conflicts of interest. K. Li, P. Szapary, and C. Brodmerkel are employees of Janssen Research & Development, LLC, and own stock in Johnson & Johnson, of which Janssen is a subsidiary. J. Krueger has received research grants on behalf of Rockefeller Institute from Janssen (formerly Centocor), honoraria from Janssen (Centocor), and has served as a consultant/advisory board member for Janssen (Centocor) and Amgen.

## Authors’ contribution

NM designed the study and drafted the manuscript. PS designed the study and critically reviewed the manuscript. CB acquired and analyzed the data, and critically reviewed the manuscript. KL analyzed the data and critically reviewed the manuscript. JK acquired the data and critically reviewed the manuscript. All authors reviewed and approved the final manuscript.

## Supplementary Material

Additional file 1: Table S1 Selected atherosclerotic cardiovascular disease (ASCVD) genes significantly modulated in psoriasis lesional skin (LS) biopsies compared with skin biopsies from healthy normal subjects. **Table S2.** Selected atherosclerotic cardiovascular disease (ASCVD) genes with significant expression modulation in psoriasis serum compared with normal serum. Click here for file
