# Supplementary material for: Modulation of cardiometabolic pathways in skin and serum from patients with psoriasis
Source: J Transl Med. 2013 Aug 22;11:194. doi: 10.1186/1479-5876-11-194 (PMC3765699; doi:10.1186/1479-5876-11-194)
Supplement: Additional file 1: Table S1 — Selected atherosclerotic cardiovascular disease (ASCVD) genes significantly modulated in psoriasis lesional skin (LS) biopsies compared with skin biopsies from healthy normal subjects. Table S2. Selected atherosclerotic cardiovascular disease (ASCVD) genes with significant expression modulation in psoriasis serum compared with normal serum. [file 1479-5876-11-194-S1.docx]

**Additional file 1 Table 1.** Selected atherosclerotic cardiovascular disease (ASCVD) genes significantly modulated in psoriasis lesional skin (LS) biopsies compared with skin biopsies from healthy normal subjects.

| **Gene Symbol** | **Probe Set ID** | **LS vs. normal**  **Fold Change** | **LS vs. normal**  **False Discovery Rate p-value** | **Gene Title** |
| --- | --- | --- | --- | --- |
| CCL2 | 216598_s_at | 7.98 | 3.09E-26 | chemokine (C-C motif) ligand 2 |
| CCL22 | 207861_at | 6.66 | 2.30E-19 | chemokine (C-C motif) ligand 22 |
| SOCS3 | 227697_at | 5.43 | 1.74E-22 | suppressor of cytokine signaling 3 |
| SAA1 /// SAA2 | 208607_s_at | 3.79 | 1.40E-06 | serum amyloid A1 /// serum amyloid A2 |
| STAT3 | 208992_s_at | 3.67 | 2.03E-13 | signal transducer and activator of transcription 3 (acute-phase response factor) |
| TNFAIP3 | 202643_s_at | 2.06 | 1.55E-09 | tumor necrosis factor, alpha-induced protein 3 |
| IKBKB | 209342_s_at | 2.03 | 1.31E-13 | inhibitor of kappa light polypeptide gene enhancer in B-cells, kinase beta |
| CXCL16 | 223454_at | 1.93 | 6.44E-21 | chemokine (C-X-C motif) ligand 16 |
| SOCS1 | 210001_s_at | 1.75 | 0.0011 | suppressor of cytokine signaling 1 |
| FAS | 215719_x_at | 1.65 | 0.0002 | Fas (TNF receptor superfamily, member 6) |
| RIPK2 | 209545_s_at | 1.53 | 3.06E-06 | receptor-interacting serine-threonine kinase 2 |
| AKT2 | 226156_at | -1.84 | 6.34E-12 | v-akt murine thymoma viral oncogene homolog 2 |
| AKT1 | 207163_s_at | -1.93 | 6.01E-08 | v-akt murine thymoma viral oncogene homolog 1 |
| SOCS2 | 203373_at | -2.25 | 6.64E-09 | suppressor of cytokine signaling 2 |
| DDIT3 /// NR1H3 | 209383_at | -2.44 | 8.01E-18 | DNA-damage-inducible transcript 3 /// nuclear receptor subfamily 1, group H, member 3 |
| FLOT1 | 213819_s_at | -4.88 | 1.27E-16 | Flotillin 1 |
| ADIPOQ | 207175_at | -5.26 | 0.0002 | adiponectin, C1Q and collagen domain containing |
| NR1H3 | 203920_at | -5.94 | 2.58E-24 | nuclear receptor subfamily 1, group H, member 3 |
| FABP4 | 235978_at | -6.45 | 3.02E-06 | fatty acid binding protein 4, adipocyte |
| PIK3R1 | 212249_at | -7.09 | 4.28E-22 | phosphoinositide-3-kinase, regulatory subunit 1 (alpha) |
| NTRK2 | 221795_at | -9.66 | 3.25E-13 | neurotrophic tyrosine kinase, receptor, type 2 |
| PPARA | 223437_at | -10.98 | 6.44E-28 | peroxisome proliferator-activated receptor alpha |
| APOC1 | 204416_x_at | -11 | 6.94E-12 | apolipoprotein C-I |
| LEP | 207092_at | -11.42 | 7.68E-10 | leptin |

**Additional file 1 Table 2.** Selected atherosclerotic cardiovascular disease (ASCVD) genes with significant expression modulation in psoriasis serum compared with normal serum.

| **Name** | **Psoriasis vs. normal**  **Fold Change** | **Psoriasis vs. normal**  **False Discovery Rate p-value** |
| --- | --- | --- |
| CD40_Ligand | 21.5498 | 0 |
| Myeloperoxidase | 6.96581 | 0 |
| MCP-1 | 3.55367 | 0 |
| PAI1 | 3.21336 | 0 |
| MDC | 3.02965 | 0 |
| TNF-α | 2.50071 | 0 |
| IL-18 | 1.84743 | 1.28E-25 |
| Apolipoprotein_A1 | -1.54639 | 9.65E-23 |
| IFN-γ | -1.81829 | 0.000114123 |
| Apolipoprotein_CIII | -2.0123 | 8.07E-05 |

Notes: Additional genes measured without significant modulation: Leptin, TNF-β, MMP9, IL-6, PAPPA, and Adiponectin
